# Emerging trends and disparities in cardiovascular, kidney, and diabetes-related mortality: A retrospective analysis of the wide-ranging online data for epidemiologic research database

**DOI:** 10.1371/journal.pone.0320670

**Published:** 2025-05-05

**Authors:** Aman Goyal, Humza Saeed, Samia Aziz Sulaiman, Wania Sultan, Momina Riaz Siddiqui, Mah I. Kan Changez, Arman Qamar, Sarju Ganatra, Sourbha S. Dani

**Affiliations:** 1 Department of Internal Medicine, Seth GS Medical College and KEM Hospital, Mumbai, India; 2 Department of Internal Medicine, Rawalpindi Medical University, Rawalpindi, Pakistan; 3 University of Jordan, School of Medicine, Jordan; 4 Department of Internal Medicine, Dow University of Health Sciences, Karachi, Pakistan; 5 Department of Cardiothoracic Surgery, Yale University, New Haven, Connecticut, United States of America; 6 Division of Interventional Cardiology and Vascular Medicine, NorthShore University Health, System, University of Chicago Pritzker School of Medicine, Evanston, Illinois, United States of America; 7 Division of Cardiovascular Medicine, Lahey Hospital and Medical Center, Beth Israel Lahey Health, Burlington, Massachusetts, United States of America; University of Calgary, CANADA

## Abstract

**Introduction:**

Cardiovascular-kidney-metabolic (CKM) syndrome, driven by metabolic risk factors like obesity, type 2 diabetes (DM-2), chronic kidney disease (CKD), and cardiovascular disease (CVD), leads to poorer health outcomes. Despite its rising prevalence and promising new therapies, trends and demographic disparities in CKM-related mortality among adults in the United States remain underexplored.

**Methodology:**

The study examined CDC WONDER death certificates for individuals aged 25+ who died from 1999 to 2022, with CVD as the main cause, while CKD and DM-2 as contributing factors. Age-adjusted mortality rates (AAMRs) and annual percent change (APC) were calculated by year, sex, age, race/ethnicity, region, and urbanization status.

**Results:**

From 1999 to 2022, 25,980 CKM-related deaths were recorded, with the AAMR decreasing from 5.3 to 0.4 per 1,000,000 population. AAMR rose significantly from 1999 to 2012 (APC: 7.03; p<0.001), sharply declined from 2012 to 2015 (APC: -65.55; p<0.001), and then increased from 2015 to 2022 (APC: 15.98; p = 0.101). Men had higher AAMRs than women (6.9 vs. 4.3), and older adults (65+) had the highest AAMR (23.3), followed by middle-aged adults (2.2). Among racial groups, non-Hispanic (NH) American Indian/Alaska Native had the highest AAMR (11.2), followed by NH Black (8.6), Hispanic (6.6), NH White (4.8), and NH Asian/Pacific Islander (4.7). Rural areas showed the highest AAMRs (6.8), compared to medium-small metro (6.1) and large metro areas (4.4).

**Conclusions:**

CKM-related mortality trends have varied widely over the past two decades, with men, older adults, American Indian/Alaska Native, and non-metropolitan populations experiencing the highest AAMRs, underscoring the need for targeted interventions.

## Introduction

The interrelationship between cardiovascular disease (CVD), chronic kidney disease (CKD), obesity, and type 2 diabetes (T2DM) is well-established through complex pathophysiological mechanisms, often referred to as the cardiovascular-kidney-metabolic (CKM) syndrome [1]. These conditions share common risk factors, such as obesity, hypertension, and dyslipidemia, which exacerbate each other’s progression [2].

More than 25% of the United States adult population was affected by CKM syndrome from 2015 to 2020. Moreover, it became the leading cause of mortality in the United States in 2021 [3]. Among individuals with both diabetes and CKD, heart failure and atherosclerotic cardiovascular disease are the primary causes of death due to increases in the risk of major atherosclerotic vascular and HF events and cardiovascular death [1,4,5]. Additionally, in the United States, diabetes and hypertension account for approximately 75% of kidney failure cases, highlighting the severe interaction between these conditions. Despite advancements in managing other diabetes complications, kidney failure cases linked to diabetes continue to rise [1,6]. Globally, deaths from CKD due to diabetes increased by 106% between 1990 and 2013 [7].

Thus, the coexistence of these pathologies significantly worsens health outcomes, leading to increased morbidity and mortality. Patients with these comorbidities face a compounded risk of adverse cardiovascular events and renal decline due to shared inflammatory pathways and hemodynamic stressors [2,3,8]. For instance, individuals with T2DM and CKD exhibit a two- to threefold increase in cardiovascular mortality compared to those without these conditions [2,8,9]. This synergistic interaction not only escalates the severity of each disease but also complicates treatment strategies, necessitating integrated management approaches [10].

Given the increasing prevalence and the impact of CKM syndrome on patient outcomes, we aim to explore emerging trends in mortality associated with these comorbid conditions among United States adults. We also aim to analyze disparities related to sex, race, and geographic distribution. Understanding these patterns is crucial for developing targeted interventions and improving clinical outcomes in populations disproportionately affected by these interlinked diseases.

## Methods

### Study setting and population

We used mortality data extracted on October 10, 2024 from the Centers for Disease Control and Prevention’s WONDER (Wide-Ranging Online Data for Epidemiologic Research) database [11]. We investigated mortality rates among adults with CKM syndrome between 1999 and 2022 (for overall AAMR and up to 2020 for further stratification). We focused on the Multiple Cause-of-Death Public Use Record database to identify cases where acute CVD was listed as the underlying cause and both T2DM and CKD were listed as the contributing causes on death certificates across the United States [12]. This database and method have been previously utilized in various CDC WONDER analysis papers [13,14]. Cases of CKM syndrome were identified using the International Statistical Classification of Diseases and Related Health Problems, 10th Revision codes: I00-I99 was used for CVD (as the underlying cause of death), E11 for T2DM, and N18 for CKD (with T2DM and CKD as multiple causes of death, using the Boolean operator ‘AND’) [15]. We did not include the ICD-10 code for obesity, as the CDC Wonder database limits the number of conditions added using the Boolean operator ‘AND’ to two. Our study was exempt from institutional review board approval, as we utilized a de-identified government-provided public-use dataset following Strengthening the Reporting of Observational Studies in Epidemiology (STROBE) guidelines [16].

### Data abstraction

Our demographic variables were defined based on population size, age distribution, sex composition, racial/ethnic background, geographic location, urbanization level, and place of death from 1999 to 2020. The locations of death included inpatient facilities, outpatient clinics, emergency rooms, cases of sudden death, residences, hospice/nursing homes, long-term care facilities, and instances with unspecified locations. Racial and ethnic groups were categorized as Hispanic (Latino), Non-Hispanic (NH) White, NH Black, NH American Indian/Alaskan Native, and NH Asian. These classifications align with those used in previous analyses from the CDC WONDER database and follow data reporting standards outlined by the United States Office of Budget and Management Guidelines, as recorded on death certificates [12].

Patients were also segmented into ten-year age intervals, categorized as young adults (25–44 years), middle-aged adults (45–64 years), and older individuals (65–85+ years), following the age distribution criteria used in previous studies with CDC WONDER data [17]. Geographical stratification was based on the Urban-Rural Classification Scheme from the National Center for Health Statistics, dividing counties into urban (large metropolitan areas with populations over 1 million), medium/small metropolitan areas (populations between 50,000 and 999,999), and rural/non-metropolitan areas (populations under 50,000). Additionally, the United States was divided into four regions according to the United States Census Bureau’s classification: Northeast, Midwest, South, and West [18]. To further assess the impact of the COVID-19 pandemic, the average AAMR from 1999 to 2019 (prior to the pandemic) was compared with that during the pandemic years (2020–2022).

### Statistical analysis

We analyzed sex, race, age, urbanization, and census-related patterns by calculating both crude and age-adjusted mortality rates (AAMR) per 1,000,000 individuals, using the 2000 United States population as the baseline for AAMR standardization [19]. Temporal changes in mortality rates were assessed using the Joinpoint Regression Program (Version 5.0.2, National Cancer Institute) [20], fitting log-linear regression models to evaluate trends over time. Following published guidelines, Joinpoint regression was applied to detect inflection points in AAMR trends for CKM syndrome from 1999 to 2020. For datasets with 17–21 time points, the guidelines recommend identifying a maximum of three inflection points, but since our study spans 22–24 years, the software was set to detect up to four joinpoints where significant changes in trend occurred. However, fewer joinpoints could be identified if greater variability between trends was captured with fewer points. The Grid Search method (2, 2, 0), permutation test, and parametric method were used to calculate annual percent change (APC) and 95% confidence intervals (CIs). An APC was considered increasing or decreasing if the slope representing the mortality change was significantly different from 0, based on 2-tailed t-tests. Statistical significance was set at P ≤ 0.05.

## Results

Between 1999 and 2022, there were 25,980 CKM syndrome-related deaths among adult patients in the United States (S1 Table). The place of death was recorded for 24,797 cases: 50.13% occurred in medical facilities, 20.9% in decedents’ homes, 22.5% in nursing homes or long-term care facilities, and 1.9% in hospices (S2 Table).

### Demographic trends in mortality

**Fig 1:**
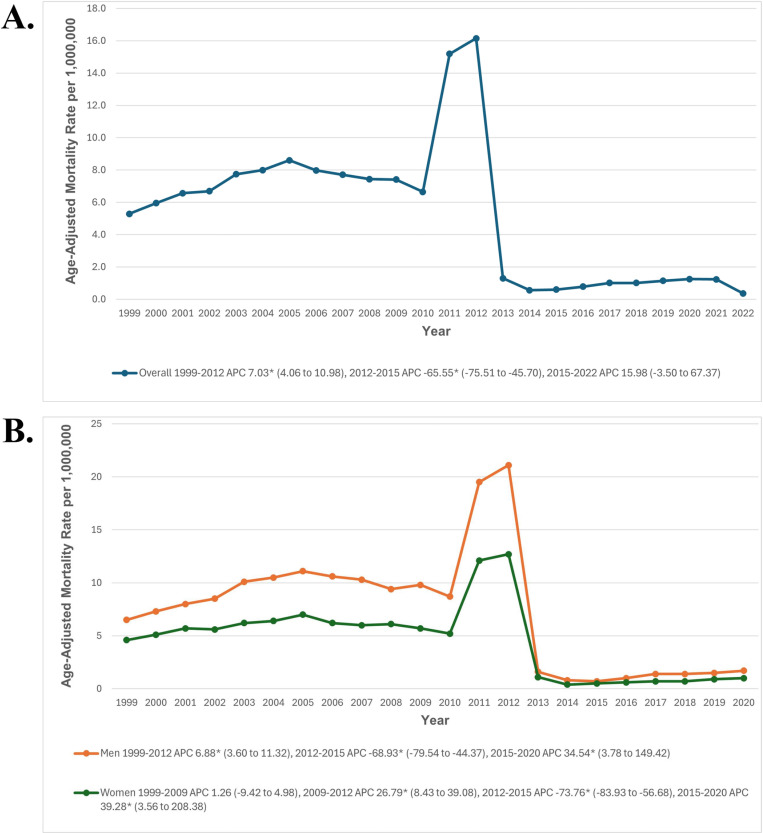
(A) Overall cardiovascular-kidney-metabolic syndrome-related Age-Adjusted Mortality Rates per 1,000,000 in Adults in the United States, 1999 to 2022, and (B) Sex-Stratified cardiovascular-kidney-metabolic syndrome-related Age-Adjusted Mortality Rates per 1,000,000 in Adults in the United States, 1999 to 2020. * Indicates that the APC is significantly different from zero at α = 0.05. AAMR = age-adjusted mortality rate; APC = annual percent change; CI = confidence interval.

The overall CKM-related AAMR was 5.3 in 1999, decreasing to 0.4 in 2022 per 1,000,000 individuals. The AAMR significantly increased from 1999 to 2012 (APC: 7.03; 95% CI: 4.06 to 10.98; p>0.001), followed by a significant decrease from 2012 to 2015 (APC: -65.55; 95% CI: -75.51 to -45.70; p>0.001). After 2015, there was a non-significant increase through 2022 (APC: 15.98; 95% CI: -3.50 to 67.37; p = 0.101) (The trends in overall CKM-related mortality and sex stratification are detailed in Fig 1, S1 and S3 Tables).

### Sex stratification

Throughout the study period, men consistently had higher AAMRs than women (6.9 vs. 4.3). Among men, the AAMR significantly increased from 1999 to 2012 (APC: 6.88; 95% CI: 3.48 to 11.34; p = 0.001), followed by a significant decrease from 2012 to 2015 (APC: -68.93; 95% CI: -79.28 to -44.09; p = 0.002). A significant uptrend was observed from 2015 to 2020 (APC: 34.54; 95% CI: 3.33 to 149.34; p = 0.029). The AAMR remained stable for women from 1999 to 2009, followed by a significant increase from 2009 to 2012 (APC: 26.79; 95% CI: 8.41 to 38.96; p = 0.008). This was followed by a significant decrease from 2012 to 2015 (APC: -73.76; 95% CI: -83.82 to -56.77; p = 0.007) and a significant uptrend from 2015 to 2020 (APC: 39.28; 95% CI: 3.52 to 207.82; p = 0.032) (Fig 1, S3 and S4 Tables).

### Stratification by age groups

**Fig 2:**
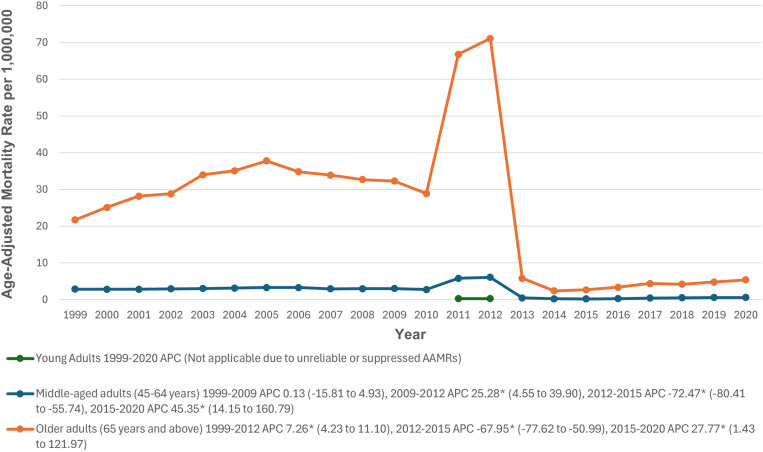
Cardiovascular-kidney-metabolic syndrome-related Age-Adjusted Mortality Rates per 1,000,000, Stratified by age groups in Adults in the United States, 1999 to 2020. * Indicates that the APC is significantly different from zero at α = 0.05. AAMR = age-adjusted mortality rate; APC = annual percent change; CI = confidence interval.

When stratified by age, older adults had the highest AAMRs (23.3), followed by middle-aged adults (2.2) and young adults (0.1). Trend analysis in young adults was not feasible due to the low number of deaths and negligible AAMR values. The AAMR remained stable among middle-aged adults from 1999 to 2009, followed by a significant increase from 2009 to 2012 (APC: 25.28; 95% CI: 4.55 to 39.90; p = 0.032). A significant decrease was observed from 2012 to 2015 (APC: -72.47; 95% CI: -80.41 to -55.74; p = 0.022), with a significant uptrend from 2015 to 2020 (APC: 45.35; 95% CI: 14.15 to 160.79; p = 0.015). In older adults, the AAMR significantly increased from 1999 to 2012 (APC: 7.26; 95% CI: 4.23 to 11.10; p>0.001), followed by a significant decrease from 2012 to 2015 (APC: -67.95; 95% CI: -77.62 to -50.99; p>0.001), and a significant uptrend from 2015 to 2020 (APC: 27.77; 95% CI: 1.43 to 121.97; p = 0.039) (The trends for CKM-related mortality after stratification by age group are detailed in Fig 2, S3 and S5 Tables).

### Racial stratification

**Fig 3:**
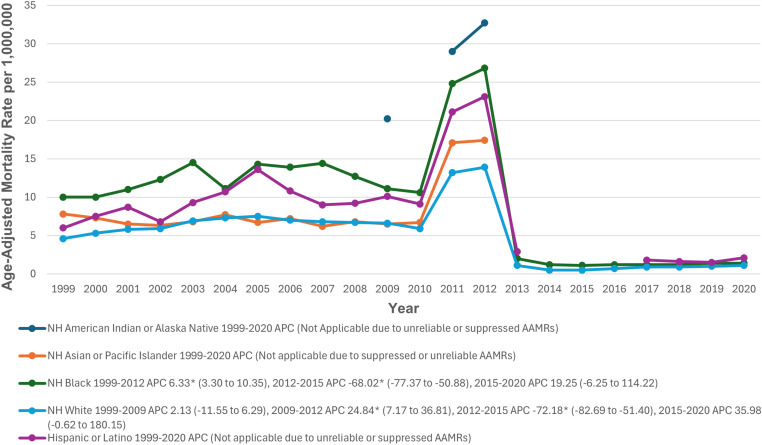
Cardiovascular-kidney-metabolic syndrome-related Age-Adjusted Mortality Rates per 1,000,000, Stratified by ethnoracial groups in Adults in the United States, 1999 to 2020. * Indicates that the APC is significantly different from zero at α = 0.05. AAMR = age-adjusted mortality rate; APC = annual percent change; CI = confidence interval.

Stratified by race/ethnicity, AAMRs were highest among NH American Indian or Alaska Native (11.2), followed by NH Black (8.6), Hispanic or Latino (6.6), NH White (4.8), and NH Asian or Pacific Islander (4.7) populations. Trend analysis for NH American Indian or Alaska Native, NH Asian or Pacific Islander, and Hispanic populations was not possible due to low numbers of deaths. Among NH Black adults, the AAMR significantly increased from 1999 to 2012 (APC: 6.33; 95% CI: 3.25 to 10.32; p>0.001), followed by a significant decline from 2012 to 2015 (APC: -68.02; 95% CI: -77.29 to -50.66; p>0.001), with a non-significant increase through 2020 (APC: 19.25; 95% CI: -6.09 to 117.23; p = 0.126). Among NH White adults, the AAMR remained stable from 1999 to 2009, followed by a significant increase from 2009 to 2012 (APC: 24.84; 95% CI: 7.02 to 36.69; p = 0.022), a significant decline from 2012 to 2015 (APC: -72.18; 95% CI: -82.36 to -51.27; p = 0.020), and a non-significant increase through 2020 (APC: 35.98; 95% CI: -1.14 to 181.12; p = 0.056) (The trends for CKM-related mortality after stratification by race are detailed in Fig 3, S3 and S6 Tables).

### State-wise distribution

**Fig 4:**
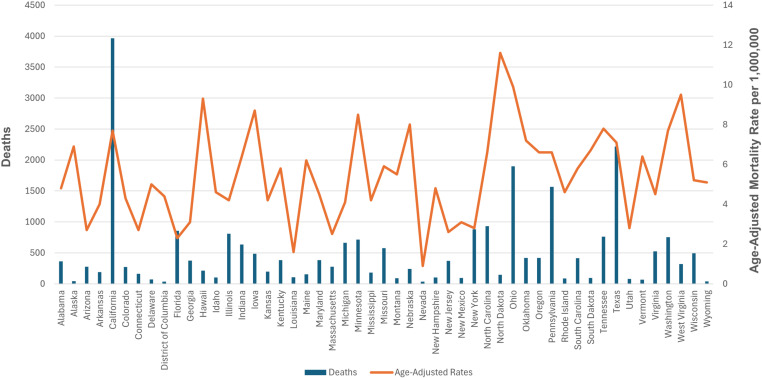
Cardiovascular-kidney-metabolic syndrome-related Age-Adjusted Mortality Rates per 1,000,000, Stratified by State in Adults in the United States, 1999 to 2020.

AAMR values varied significantly by state, ranging from 0.9 in Nevada to 11.6 in North Dakota. States in the top 90th percentile (Minnesota, Iowa, Hawaii, West Virginia, Ohio, North Dakota) had AAMRs approximately four times higher than those in the bottom 10th percentile (Nevada, Louisiana, Florida, Massachusetts, New Jersey, Connecticut, Arizona) (The trends for CKM-related mortality after stratification by states are detailed in Fig 4, S7 Table).

### Census region

**Fig 5:**
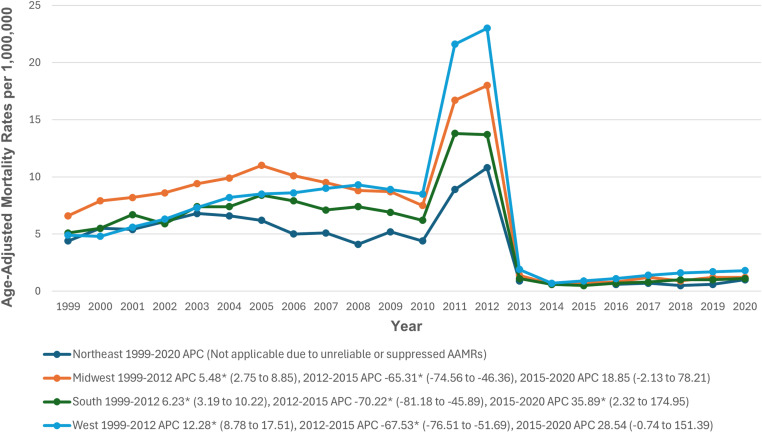
Cardiovascular-kidney-metabolic syndrome-related Age-Adjusted Mortality Rates per 1,000,000, Stratified by Census Regions in Adults in the United States, 1999 to 2020. * Indicates that the APC is significantly different from zero at α = 0.05. AAMR = age-adjusted mortality rate; APC = annual percent change; CI = confidence interval.

During the study period, the highest AAMR values were in the Midwestern region (6.4), followed by the Western (6.3), Southern (4.9), and Northeastern (3.9) regions. Trend analysis for the Northeastern region was impossible due to unreliable AAMR values. In the Southern region, AAMRs significantly increased from 1999 to 2012 (APC: 6.23; 95% CI: 3.19 to 10.22; p = 0.001), followed by a significant decline from 2012 to 2015 (APC: -70.22; 95% CI: -81.18 to -45.89; p = 0.004), and a significant increase through 2020 (APC: 35.89; 95% CI: 2.32 to 174.95; p = 0.037). Similar trends were observed in the Midwestern region, with a significant increase from 1999 to 2012 (APC: 5.48; 95% CI: 2.75 to 8.85; p>0.001), a significant decline from 2012 to 2015 (APC: -65.31; 95% CI: -74.56 to -46.36; p>0.001), and a non-significant increase through 2020 (APC: 18.85; 95% CI: -2.13 to 78.21; p = 0.077). In the Western region, there was a significant increase from 1999 to 2012 (APC: 12.28; 95% CI: 8.78 to 17.51; p>0.001), followed by a significant decline from 2012 to 2015 (APC: -67.53; 95% CI: -76.51 to -51.69; p>0.001), and a non-significant increase through 2020 (APC: 28.53; 95% CI: -0.74 to 151.39; p = 0.057) (The trends for CKM-related mortality after stratification by census region are detailed in Fig 5, S3 and S8 Tables).

### Urbanization

**Fig 6:**
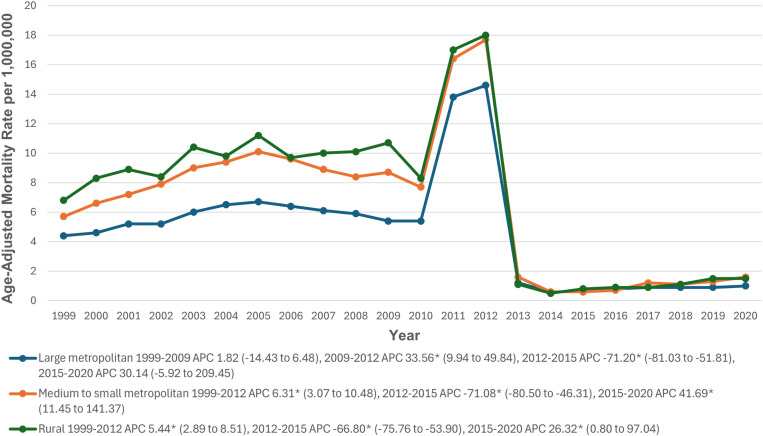
Cardiovascular-kidney-metabolic syndrome-related Age-Adjusted Mortality Rates per 1,000,000 in Adults in the Metropolitan and Non-metropolitan areas in the United States, 1999 to 2020. * Indicates that the APC is significantly different from zero at α = 0.05. AAMR = age-adjusted mortality rate; APC = annual percent change; CI = confidence interval.

Throughout the study period, non-metropolitan/rural areas had the highest AAMRs (6.8), followed by medium to small metropolitan areas (6.1) and large metropolitan areas (4.4). In non-metropolitan/rural areas, the AAMR significantly increased from 1999 to 2012 (APC: 7.35; 95% CI: 4.36 to 11.08; p>0.001), followed by a significant decrease from 2012 to 2015 (APC: -65.82; 95% CI: -75.70 to -45.64; p>0.001), and a non-significant increase through 2020 (APC: 12.46; 95% CI: -1.14 to 49.36; p = 0.070). Similar trends were observed in medium to small metropolitan areas, with a significant increase from 1999 to 2012 (APC: 5.34; 95% CI: 2.21 to 9.68; p=0.002), a significant decrease from 2012 to 2015 (APC: -61.79; 95% CI: -72.92 to -37.22; p=0.002), and a non-significant increase through 2020 (APC: 21.65; 95% CI: -3.28 to 131.23; p=0.084). In large metropolitan areas, the AAMR significantly increased from 1999 to 2012 (APC: 6.36; 95% CI: 3.12 to 10.74; p>0.001), followed by a significant decrease from 2012 to 2015 (APC: -67.46; 95% CI: -77.26 to -48.32; p>0.001), and a non-significant increase through 2020 (APC: 15.89; 95% CI: -4.26 to 73.28; p=0.097) (The trends for CKM-related mortality after stratification by urbanization level are detailed in Fig 6, S3 and S9 Tables).

### Trends in mortality rates by individual disease type

#### Mortality trends in cardiovascular disease (CVD).

**Fig 7:**
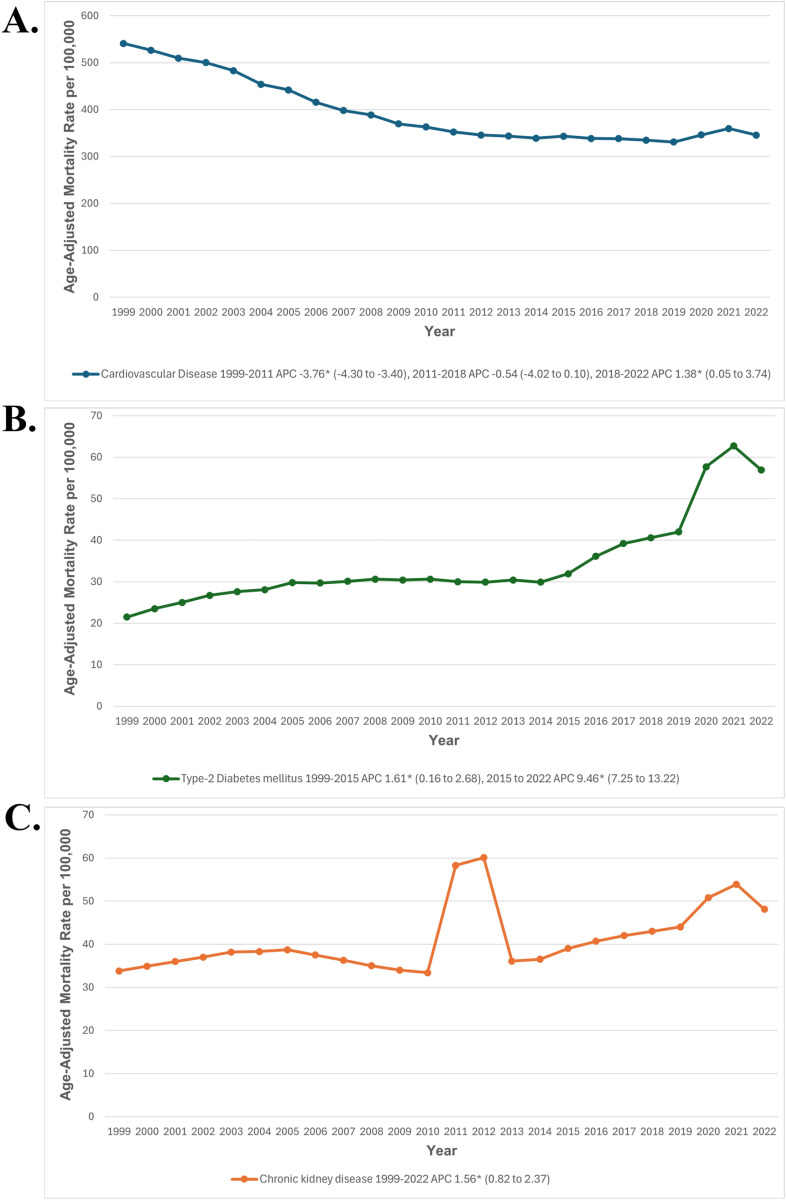
(A) Cardiovascular Disease and (B) type-2 Diabetes Mellitus, and (C) chronic kidney disease- related Age-Adjusted Mortality Rates per 100,000 in Adults in the United States, 1999 to 2022. * Indicates that the APC is significantly different from zero at α = 0.05. AAMR = age-adjusted mortality rate; APC = annual percent change; CI = confidence interval.

The AAMR for CVD among United States adults was 541 in 1999, decreasing to 345.6 per 100,000 individuals in 2022. The AAMR significantly declined from 1999 to 2011 (APC: -3.76; 95% CI: -4.30 to -3.40; p>0.001), followed by a stable period from 2011 to 2018. However, a significant increase was observed from 2018 to 2022 (APC: 1.38; 95% CI: 0.05 to 3.74; p=0.041) (Fig 7A, S3 and S10 Tables).

#### Mortality trends in type-2 diabetes mellitus alone.

The AAMR for T2DM among United States adults was 21.5 in 1999, rising to 56.9 per 100,000 individuals in 2022. From 1999 to 2015, AAMR values increased significantly (APC: 1.61; 95% CI: 0.16 to 2.68; p=0.034), followed by a steep uptrend from 2015 to 2022 (APC: 9.46; 95% CI: 7.25 to 13.22; p>0.001) (Fig 7B, S3 and S10 Tables).

#### Mortality trends in chronic kidney disease alone.

The AAMR for CKD among United States adults was 33.8 in 1999, increasing to 48.1 per 100,000 individuals in 2022. AAMR values showed a significant rise over the study period from 1999 to 2022 (APC: 1.56; 95% CI: 0.82 to 2.37; p>0.001) (Fig 7C, S3 and S10 Tables).

### Trend comparison before and after the COVID-19 pandemic

**Fig 8:**
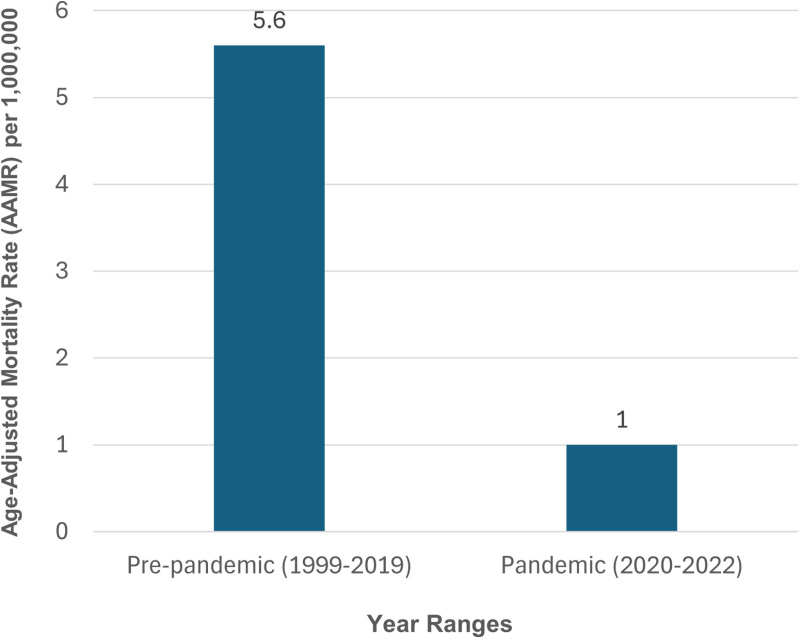
Comparison of Cardiovascular-kidney-metabolic syndrome-related-related Average Age-Adjusted Mortality Rates (AAMRs) per 1,000,000 during the COVID-19 Pandemic period (2020-2022) with the pre-Pandemic period (1999-2019).

The AAMR values increased from 1.1 per million in 2019 to 1.3 in 2020 and 1.2 in 2021 and decreased to 0.4 towards the end of the pandemic in 2022. Despite this increase, the overall AAMR value for the pandemic period of 2020–2022 (1.0 per million) was significantly lower than the overall AAMR in the pre-pandemic period of 1999–2019 (5.6 per million) (Fig 8, S1 Table).

## Discussion

Our findings demonstrate that between 1999 and 2022, CKM-related deaths among United States adults totaled 25,980, with 50% occurring in medical facilities. Following a significant increase in the AAMR from 1999 to 2012 (APC: 7.03; p<0.001), a significant decrease from 2012 to 2015 (APC: -65.55; p<0.001), and a non-significant increase since 2015 (APC: 15.98; p=0.101), the AAMR for CKM has significantly reduced from 5.3 per million in 1999 to 0.4 in 2022. Moreover, multiple demographic trends have been found. Men consistently had higher AAMRs than women (AAMR: 6.9 vs. 4.3) and older adults had the highest rates (AAMR: 23.3) regardless of sex. AAMR also varied by race, with the highest rates noticed amongst NH American Indian or Alaska Native (AAMR: 11.2), while the lowest in NH Asian or Pacific Islander populations (AAMR: 4.7). Geographic variability was also observed, with states like North Dakota having the highest rates and Nevada the lowest (AAMR: 11.6 vs. 0.9). Additionally, rural areas had the highest AAMR among the urbanization levels (AAMR: 6.8), highlighting significant disparities in CKM-related mortality across different settings.

The overall decrease in AAMR from 1999 to 2022 could be explained by increased access to healthcare and the availability of treatment. Despite AAMR decreasing over time, studies demonstrate that cardiovascular, renal, and metabolic disease prevalence is increasing over time [21,22]. Moreover, CKM syndrome was identified as the leading cause of death in the United States in 2021 [3]. A study conducted by Ong et al. found that significant food system changes, including increased use of shelf-stable, high-calorie, and ultra-processed, and fat, sugar, and animal products, limited financial and proximal access to healthy food, and decrease in physical activity related to global work and transportation trends, all contribute to increasing BMI, which is associated increased prevalence of T2DM since 1990 [21]. They also highlight that T2DM may be preventable and potentially reversible if managed early on, which necessitates increasing awareness and screening programs. Therefore, the significant increase in AAMR from 1999 to 2012 may be related to increased diagnoses and healthcare awareness, yet it might also be due to increased unhealthy lifestyle habits. Similarly, Roth et al. have demonstrated that increased smoking, lower physical activity, and increased obesity rates have been associated with rising rates of CVD and risk of mortality from 1990 to 2019 [22]. The significant decrease in AAMR between 2012 and 2015 may be attributed to increased access to healthcare, which has been documented to improve in the United States between 2014 and 2017 [23]. This may have improved the quality of care and thus offered better management strategies to patients, especially those at high risk of complications, contributing to decreasing mortality rates. A study by Sud et al. has similarly found that there have been marked advances in understanding cardiovascular and CKD in 2014 and 2015, which could have also been a reason for the decrease in AAMR despite the increasing prevalence of those diseases [24]. We also hypothesize that in the past, patients may have also been presented later in the course of the disease, which might have increased mortality rates. Mensah et al. support such a hypothesis as they have found that improvements in prevention, early detection, treatment, and control of CVD and risk factors altogether decrease mortality rates [25]. However, they elaborate that the decline in CVD mortality has decelerated with the increase in obesity, metabolic syndrome, and T2DM in the past 20 years since 1990, consistent with our findings [25].

Regarding demographic trends, AAMR is believed to be higher amongst older patients than younger ones, likely due to the higher prevalence of comorbidities that may complicate CKM in the older population. Jaradat et al. highlight that individual risk factors and comorbidities must be considered in managing CKM [26]. As for differences between men and women, women have been found to have significantly lower AAMR than men. Similarly, in adults with CKD, women have decreased cardiovascular events, cardiovascular mortality, and overall mortality compared to men. However, there is a lower prevalence in men compared to females, and the prognosis among male patients is poorer [27]. Yu et al.‘s study indicates that CKD is more prevalent in postmenopausal women than in men and premenopausal women, which may suggest a potential protective role of estrogen in kidney function [28]. This aligns with findings from animal studies that propose estrogen’s renoprotective effects, including its influence on the synthesis, function, and degradation of matrix components, modulation of cytokines, reactive oxygen species (ROS), and vasoactive agents, all of which can cumulatively impact kidney function and metabolism [29–31]. However, it is important to note that while these mechanisms are well-documented in animal models, further research is needed to confirm their applicability and extent in humans.

As for racial and geographic trends, they are likely to be associated with the social determinants of health, as shown by Ndumele et al., where some ethnic groups, such as black individuals and those who live with lower socioeconomic status, are more likely to have higher AAMRs concerning populations that have better socioeconomic conditions [1]. However, these differences could also be related to ancestry, where a higher burden of metabolic risk factors among people of South Asian ancestry is likely associated with a higher degree of ectopic fat deposition at a given body mass index, putting them at higher risk to develop certain conditions [1]. The study highlights that epigenetic changes, driven by the interplay of genetic, environmental, social, and lifestyle factors, may help explain the biological basis for the varied manifestations of CKM. This suggests that while socioeconomic factors are significant, they are not the sole contributors to the racial and geographical differences in AAMR.

### Limitations

Our study has a few limitations to report. First, we used multiple cause-of-death methods to extract cases where CKD and T2DM were listed as contributing causes, while CVD was the underlying cause. The use of the Boolean operator ‘AND’ to pool CKD and T2DM limited our ability to include metabolic syndrome and obesity as causes of death. This limitation is inherent to the database, as more than two ICD-10 codes cannot be combined if the ‘AND’ operator is used. Second, since the ‘AND’ operator was used to combine the pathologies to fit the definition of CKM syndrome, if any of the pathologies (CVD, CKD, or DM) was missing from the death certificate, it would not meet our inclusion criteria. This is the primary reason why the total number of deaths over the past two decades was lower than the actual prevalence. Moreover, during the initial years of the analysis (1999 to the early 2000s), certain concepts, such as CKD, were not well-defined, which could impact the robustness of the analysis for those years. However, to preserve important temporal differences and provide a comprehensive overview, the data was included despite this limitation. Third, relying solely on mortality data may overlook the significant morbidity associated with CKM syndrome, as non-fatal cases can greatly affect quality of life and healthcare utilization. Fourth, the ICD-10 codes in the CDC WONDER dataset provide diagnostic information but lack detailed clinical data or patient characteristics. While these codes offer a broad picture of a patient’s condition, they may omit key factors such as comorbidities, disease severity, treatment regimens, socioeconomic status, and healthcare access—all of which play a vital role in mortality outcomes. Moreover, the dataset does not include information on previous cardiovascular, renal, or metabolic disease history, limiting our understanding of the underlying conditions contributing to CKM-related deaths. Fourth, relying on the CDC WONDER database, which may include inconsistencies in mortality data due to variations in coding and reporting practices over time, could impact the accuracy of our findings. Lastly, the CDC WONDER database does not provide baseline characteristics of patients or individual-level data, which limits the ability to perform regression or matched analyses to mitigate the risk of confounding bias.

## Conclusions

With the recent recognition of CKM as a distinct syndrome in 2023, our study highlights important trends and disparities in mortality among these patients in the United States. A concerning deceleration in the decline of mortality is evident, with men, older adults, NH American Indian/Alaska Native individuals, followed by Black adults, facing the highest risk. Geographic disparities are also prominent, with rural and Midwestern areas being most affected. These findings emphasize the urgent need for public health action and targeted interventions to reduce disparities.

## Supporting information

S1 Table Overall Cardiovascular-kidney metabolic syndrome-related Mortality per 1,000,000 Adults in the United States, 1999–2022. (DOCX)

S2 Table Cardiovascular-kidney metabolic syndrome -related Mortality, Stratified by Place of Death per 1,000,000 Adults in the United States, 1999–2020. (DOCX)

S3 Table Annual percent change (APC) of Cardiovascular-kidney metabolic syndrome–related Age-Adjusted Mortality Rates per 1,000,000 in Adults in the United States, 1999–2020. APC = Annual percent change; NH = non-Hispanic. N/A = unreliable or suppressed.
(DOCX)

S4 Table Cardiovascular-kidney metabolic syndrome -related Mortality, Stratified by Sex per 1,000,000 Adults in the United States, 1999–2020. (DOCX)

S5 Table Cardiovascular-kidney metabolic syndrome-related Mortality per 1,000,000, Stratified by Age group in Adults in the United States, 1999–2020. Young Adult = 25–44 years; Middle Aged Adults = 45–64 years; Older Adults = 65 years and above; N/A = unreliable or suppressed.
(DOCX)

S6 Table Cardiovascular-kidney metabolic syndrome -related Age-Adjusted Mortality Rates per 1,000,000, Stratified by Race in Adults in the United States from 1999 to 2020. NH=non-Hispanic; N/A = unreliable or suppressed.
(DOCX)

S7 Table Cardiovascular-kidney metabolic syndrome -related Age-Adjusted Mortality Rates per 1,000,000, Stratified by State in Adults in the United States, 1999–2020. (DOCX)

S8 Table Cardiovascular-kidney metabolic syndrome -related Age-Adjusted Mortality Rate per 1,000,000, Stratified by Census Region in Adults in the United States 1999–2020. (DOCX)

S9 Table Overall Cardiovascular-kidney metabolic syndrome –related Age-Adjusted Mortality Rates per 1,000,000, Stratified by Urbanization Status in Adults in the United States, 1999–2020. (DOCX)

S10 Table Overall Individual disease–related Age-Adjusted Mortality Rates per 100,000 in the United States, 1999–2020. (DOCX)

## Abbreviations

APC: Annual percentage change
AAMR: Age-adjusted mortality rates
NH: Non-Hispanic
CKM: Cardiovascular-kidney-metabolic
DM: Type 2 Diabetes Mellitus
CVD: Cardiovascular disease
CKD: Chronic kidney disease
