# Expression of Concern: Emerging trends and disparities in cardiovascular, kidney, and diabetes-related mortality: A retrospective analysis of the wide-ranging online data for epidemiologic research database

**DOI:** 10.1371/journal.pone.0346397

**Published:** 2026-04-06

**Authors:** The PLOS One Editors

The *PLOS One* Editors issue this Expression of Concern to alert readers that this article [1] was identified as one of a series of submissions for which we have concerns about authorship and adherence to the journal’s first and second publication criteria [2]. We regret that these issues were not addressed prior to the article’s publication.

## References

[1] Goyal A, Saeed H, Sulaiman SA, Sultan W, Siddiqui MR, Kan Changez MI, et al. Emerging trends and disparities in cardiovascular, kidney, and diabetes-related mortality: a retrospective analysis of the wide-ranging online data for epidemiologic research database. PLoS One. 2025;20(5):e0320670. doi: 10.1371/journal.pone.0320670 40323906 PMC12052136

[2] PLOS One. Criteria for Publication [Internet] Available from: https://journals.plos.org/plosone/s/criteria-for-publication [Accessed March 2026]

