# Supplementary material for: Emerging trends and disparities in cardiovascular, kidney, and diabetes-related mortality: A retrospective analysis of the wide-ranging online data for epidemiologic research database
Source: PLoS One. 2025 May 5;20(5):e0320670. doi: 10.1371/journal.pone.0320670 (PMC12052136; doi:10.1371/journal.pone.0320670)
Supplement: S1 Table — (DOCX) [file pone.0320670.s001.docx]

**S1 Table. Overall Cardiovascular-kidney metabolic syndrome-related Mortality per 1,000,000 Adults in the United States, 1999 to 2022.**

| Year | Deaths | Age-Adjusted Mortality Rate (95% CI) | Population |
| --- | --- | --- | --- |
| 1999 | 934 | 5.3 (5-5.6) | 180408769 |
| 2000 | 1061 | 6 (5.6-6.3) | 181984640 |
| 2001 | 1190 | 6.6 (6.2-6.9) | 184305128 |
| 2002 | 1230 | 6.7 (6.3-7.1) | 186208028 |
| 2003 | 1447 | 7.7 (7.3-8.1) | 188090429 |
| 2004 | 1517 | 8 (7.6-8.4) | 190205384 |
| 2005 | 1665 | 8.6 (8.2-9) | 192551384 |
| 2006 | 1579 | 8 (7.6-8.4) | 195019359 |
| 2007 | 1545 | 7.7 (7.3-8.1) | 197403777 |
| 2008 | 1533 | 7.4 (7.1-7.8) | 199795090 |
| 2009 | 1556 | 7.4 (7-7.8) | 202107016 |
| 2010 | 1421 | 6.7 (6.3-7) | 203891983 |
| 2011 | 3323 | 15.2 (14.7-15.7) | 206592936 |
| 2012 | 3623 | 16.2 (15.6-16.7) | 208826037 |
| 2013 | 299 | 1.3 (1.2-1.5) | 211085314 |
| 2014 | 133 | 0.6 (0.5-0.7) | 213809280 |
| 2015 | 148 | 0.6 (0.5-0.7) | 216553817 |
| 2016 | 193 | 0.8 (0.7-0.9) | 218641417 |
| 2017 | 259 | 1 (0.9-1.1) | 221447331 |
| 2018 | 261 | 1 (0.9-1.1) | 223311190 |
| 2019 | 300 | 1.1 (1-1.3) | 224981167 |
| 2020 | 343 | 1.3 (1.1-1.4) | 226635013 |
| 2021 | 322 | 1.23 (1.09-1.37) | 4473854489 |
| 2022 | 98 | 0.4 (0.3-0.4) | 180408769 |
