# Supplementary material for: Emerging trends and disparities in cardiovascular, kidney, and diabetes-related mortality: A retrospective analysis of the wide-ranging online data for epidemiologic research database
Source: PLoS One. 2025 May 5;20(5):e0320670. doi: 10.1371/journal.pone.0320670 (PMC12052136; doi:10.1371/journal.pone.0320670)
Supplement: S2 Table — (DOCX) [file pone.0320670.s002.docx]

**S2 Table. Cardiovascular-kidney metabolic syndrome -related Mortality, Stratified by Place of Death per 1,000,000 Adults in the United States, 1999 to 2020.**

| **Deaths** | | | | |
| --- | --- | --- | --- | --- |
| **Year** | **Medical**  **Facility** | **Nursing Home/Long-term**  **Care Facility** | **Hospices** | **Home** |
| 1999 | 640 | 154 | Missing | 127 |
| 2000 | 636 | 246 | Missing | 161 |
| 2001 | 710 | 259 | Missing | 188 |
| 2002 | 709 | 287 | Missing | 203 |
| 2003 | 808 | 354 | Suppressed | 246 |
| 2004 | 855 | 332 | Suppressed | 285 |
| 2005 | 882 | 420 | 13 | 306 |
| 2006 | 788 | 363 | 21 | 365 |
| 2007 | 798 | 358 | 18 | 329 |
| 2008 | 777 | 354 | 25 | 317 |
| 2009 | 773 | 359 | 38 | 315 |
| 2010 | 725 | 321 | 52 | 294 |
| 2011 | 1522 | 759 | 113 | 817 |
| 2012 | 1630 | 852 | 112 | 912 |
| 2013 | 144 | 63 | 13 | 68 |
| 2014 | 54 | 38 | Suppressed | 29 |
| 2015 | 70 | 38 | Suppressed | 36 |
| 2016 | 80 | 49 | 11 | 45 |
| 2017 | 112 | 54 | 18 | 67 |
| 2018 | 103 | 50 | 14 | 86 |
| 2019 | 109 | 69 | 13 | 94 |
| 2020 | 101 | 64 | 14 | 148 |
| **Total** | 13026 | 5843 | 490 | 5438 |
