# Supplementary material for: Emerging trends and disparities in cardiovascular, kidney, and diabetes-related mortality: A retrospective analysis of the wide-ranging online data for epidemiologic research database
Source: PLoS One. 2025 May 5;20(5):e0320670. doi: 10.1371/journal.pone.0320670 (PMC12052136; doi:10.1371/journal.pone.0320670)
Supplement: S3 Table — APC = Annual percent change; NH = non-Hispanic. N/A = unreliable or suppressed. (DOCX) [file pone.0320670.s003.docx]

**S3 Table. Annual percent change (APC) of Cardiovascular-kidney metabolic syndrome–related Age-Adjusted Mortality Rates per 1,000,000 in Adults in the United States, 1999 to 2020.**

| Year Interval | APC (95% CI) | p-value |
| --- | --- | --- |
| Overall | | |
| 1999-2012 | 7.03 (4.06 to 10.98) | <0.001 |
| 2012-2015 | -65.55 (-75.51 to -45.70) | <0.001 |
| 2015-2022 | 15.98 (-3.50 to 67.37) | 0.101 |
| Men | | |
| 1999-2012 | 6.88 (3.48 to 11.34) | 0.001 |
| 2012-2015 | -68.93 (-79.28 to -44.09) | 0.002 |
| 2015-2020 | 34.54 (3.33 to 149.34) | 0.029 |
| Women | | |
| 1999-2009 | 1.26 (-9.11 to 5.00) | 0.829 |
| 2009-2012 | 26.79 (8.41 to 38.96) | 0.008 |
| 2012-2015 | -73.76 (-83.82 to -56.77) | 0.007 |
| 2015-2020 | 39.28 (3.52 to 207.82) | 0.032 |
| Young Adults (25-44 years) | | |
| 1999-2020 | N/A | N/A |
| Middle Aged Adults (45-64 years) | | |
| 1999-2009 | 0.13 (-15.81 to 4.93) | 0.709 |
| 2009-2012 | 25.28 (4.55 to 39.90) | 0.032 |
| 2012-2015 | -72.47 (-80.41 to -55.74) | 0.022 |
| 2015-2020 | 45.35 (14.15 to 160.79) | 0.015 |
| Older Adults (65 years and above) | | |
| 1999-2012 | 7.26 (4.23 to 11.10) | <0.001 |
| 2012-2015 | -67.95 (-77.62 to -51.00) | <0.001 |
| 2015-2020 | 27.77 (1.43 to 121.97) | 0.039 |
| NH American Indian or Alaska Native | | |
| 1999-2020 | N/A | N/A |
| NH Black or African American | | |
| 1999-2012 | 6.33 (3.25 to 10.32) | <0.001 |
| 2012-2015 | -68.02 (-77.29 to -50.66) | <0.001 |
| 2015-2020 | 19.25 (-6.09 to 117.22) | 0.126 |
| Hispanic or Latino | | |
| 1999-2020 | N/A | N/A |
| NH Asian or Pacific Islander | | |
| 1999-2020 | N/A | N/A |
| NH White | | |
| 1999-2009 | 2.13 (-10.62 to 6.31) | 0.632 |
| 2009-2012 | 24.84 (7.02 to 36.69) | 0.022 |
| 2012-2015 | -73.18 (-82.36 to -51.27) | 0.020 |
| 2015-2020 | 35.98 (-1.14 to 181.12) | 0.056 |
| Large metropolitan area | | |
| 1999-2009 | 1.82 (-14.43 to 6.48) | 0.832 |
| 2009-2012 | 33.56 (9.94 to 49.84) | 0.013 |
| 2012-2015 | -71.20 (-81.03 to -51.81) | 0.013 |
| 2015-2020 | 30.14 (-5.92 to 209.45) | 0.093 |
| Medium to small metropolitan area | | |
| 1999-2012 | 6.31 (3.07 to 10.48) | <0.001 |
| 2012-2015 | -72.08 (-80.50 to -46.31) | <0.001 |
| 2015-2020 | 41.69 (11.45 to 141.37) | 0.003 |
| Nonmetropolitan area | | |
| 1999-2012 | 5.44 (2.89 to 8.51) | <0.001 |
| 2012-2015 | -66.81 (-75.76 to -53.90) | <0.001 |
| 2015-2020 | 26.33 (0.80 to 97.04 | 0.042 |
| Northeast | | |
| 1999-2020 | N/A | N/A |
| Midwest | | |
| 1999-2012 | 5.48 (2.75 to 8.85) | <0.001 |
| 2012-2015 | -65.31 (-74.56 to -46.36) | <0.001 |
| 2015-2020 | 18.85 (-2.13 to 78.21) | 0.077 |
| South | | |
| 1999-2012 | 6.23 (3.19 to 10.22) | 0.001 |
| 2012-2015 | -70.22 (-81.18 to -45.89) | 0.004 |
| 2015-2020 | 35.89 (2.33 to 174.95) | 0.037 |
| West | | |
| 1999-2002 | 12.28 (8.79 to 17.51) | <0.001 |
| 2002-2015 | -67.53 (-76.51 to -51.69) | <0.001 |
| 2015-2020 | 28.54 (-0.75 to 151.39) | 0.057 |
| Cardiovascular Disease Alone | | |
| 1999-2011 | -376 (-4.31 to -3.40) | <0.001 |
| 2011-2018 | -0.54 (-4.02 to 0.10) | 0.080 |
| 2018-2022 | 1.38 (0.05 to 3.74) | 0.041 |
| Chronic Kidney Disease Alone | | |
| 1999-2022 | 1.56 (0.83 to 2.37) | <0.001 |
| Diabetes Mellitus Type-2 Alone | | |
| 1999-2015 | 1.61 (0.16 to 2.68) | 0.034 |
| 2015-2022 | 9.46 (7.25 to 13.22) | <0.001 |

APC = Annual percent change; NH = non-Hispanic. N/A = unreliable or suppressed
