# Supplementary material for: Emerging trends and disparities in cardiovascular, kidney, and diabetes-related mortality: A retrospective analysis of the wide-ranging online data for epidemiologic research database
Source: PLoS One. 2025 May 5;20(5):e0320670. doi: 10.1371/journal.pone.0320670 (PMC12052136; doi:10.1371/journal.pone.0320670)
Supplement: S4 Table — (DOCX) [file pone.0320670.s004.docx]

**S4 Table. Cardiovascular-kidney metabolic syndrome -related Mortality, Stratified by Sex per 1,000,000 Adults in the United States, 1999 to 2020.**

| Year | Deaths | | Age-adjusted Mortality Rate (95 % CI) | |
| --- | --- | --- | --- | --- |
|  | **Women** | **Men** | **Men** | **Women** |
| 1999 | 477 | 457 | 6.5 (5.9-7.1) | 4.6 (4.2-5) |
| 2000 | 544 | 517 | 7.3 (6.6-7.9) | 5.1 (4.7-5.5) |
| 2001 | 612 | 578 | 8 (7.3-8.7) | 5.7 (5.2-6.1) |
| 2002 | 615 | 615 | 8.5 (7.8-9.1) | 5.6 (5.2-6.1) |
| 2003 | 692 | 755 | 10.1 (9.3-10.8) | 6.2 (5.8-6.7) |
| 2004 | 715 | 802 | 10.5 (9.8-11.3) | 6.4 (5.9-6.8) |
| 2005 | 804 | 861 | 11.1 (10.4-11.9) | 7 (6.5-7.5) |
| 2006 | 725 | 854 | 10.6 (9.9-11.4) | 6.2 (5.8-6.7) |
| 2007 | 707 | 838 | 10.3 (9.6-11) | 6 (5.5-6.4) |
| 2008 | 736 | 797 | 9.4 (8.7-10) | 6.1 (5.7-6.6) |
| 2009 | 708 | 848 | 9.8 (9.2-10.5) | 5.7 (5.3-6.1) |
| 2010 | 649 | 772 | 8.7 (8.1-9.3) | 5.2 (4.8-5.6) |
| 2011 | 1551 | 1772 | 19.5 (18.6-20.5) | 12.1 (11.5-12.7) |
| 2012 | 1670 | 1953 | 21.1 (20.1-22) | 12.7 (12.1-13.3) |
| 2013 | 142 | 157 | 1.6 (1.4-1.9) | 1.1 (0.9-1.2) |
| 2014 | 46 | 87 | 0.8 (0.7-1.1) | 0.4 (0.3-0.5) |
| 2015 | 71 | 77 | 0.7 (0.6-0.9) | 0.5 (0.4-0.6) |
| 2016 | 86 | 107 | 1 (0.8-1.2) | 0.6 (0.5-0.8) |
| 2017 | 104 | 155 | 1.4 (1.2-1.6) | 0.7 (0.6-0.9) |
| 2018 | 104 | 157 | 1.4 (1.2-1.7) | 0.7 (0.6-0.8) |
| 2019 | 133 | 167 | 1.5 (1.2-1.7) | 0.9 (0.7-1) |
| 2020 | 146 | 197 | 1.7 (1.5-1.9) | 1 (0.8-1.1) |
| Total | 12037 | 13523 | 6.9 (6.7-7) | 4.3 (4.2-4.4) |
