# Supplementary material for: Emerging trends and disparities in cardiovascular, kidney, and diabetes-related mortality: A retrospective analysis of the wide-ranging online data for epidemiologic research database
Source: PLoS One. 2025 May 5;20(5):e0320670. doi: 10.1371/journal.pone.0320670 (PMC12052136; doi:10.1371/journal.pone.0320670)
Supplement: S5 Table — Young Adult = 25–44 years; Middle Aged Adults = 45–64 years; Older Adults = 65 years and above; N/A = unreliable or suppressed. (DOCX) [file pone.0320670.s005.docx]

**S5 Table. Cardiovascular-kidney metabolic syndrome-related Mortality per 1,000,000, Stratified by Age group in Adults in the United States, 1999 to 2020.**

|  | **Deaths** | | | **Age-Adjusted Rate (95% CI)** | | |
| --- | --- | --- | --- | --- | --- | --- |
| **Year** | **Young Adults** | **Middle Aged Adults** | **Older Adults** | **Young Adults** | **Middle Aged Adults** | **Older Adults** |
| 1999 | Suppressed | 176 | 750 | N/A | 2.9 (2.5-3.3) | 21.7 (20.2-23.3) |
| 2000 | 10 | 176 | 875 | N/A | 2.9 (2.4-3.3) | 25.1 (23.4-26.8) |
| 2001 | 12 | 182 | 996 | N/A | 2.9 (2.4-3.3) | 28.2 (26.5-30) |
| 2002 | Suppressed | 198 | 1025 | N/A | 3 (2.6-3.4) | 28.8 (27-30.5) |
| 2003 | 10 | 211 | 1226 | N/A | 3.1 (2.6-3.5) | 34 (32.1-35.9) |
| 2004 | Suppressed | 228 | 1280 | N/A | 3.2 (2.7-3.6) | 35.1 (33.2-37) |
| 2005 | 12 | 252 | 1401 | N/A | 3.3 (2.9-3.7) | 37.8 (35.8-39.8) |
| 2006 | 10 | 260 | 1309 | N/A | 3.3 (2.9-3.7) | 34.8 (32.9-36.6) |
| 2007 | Suppressed | 236 | 1300 | N/A | 3 (2.6-3.3) | 33.9 (32-35.7) |
| 2008 | Suppressed | 250 | 1276 | N/A | 3 (2.6-3.4) | 32.7 (30.9-34.4) |
| 2009 | 11 | 260 | 1285 | N/A | 3 (2.7-3.4) | 32.3 (30.5-34.1) |
| 2010 | 11 | 242 | 1168 | N/A | 2.8 (2.4-3.1) | 28.9 (27.3-30.6) |
| 2011 | 26 | 523 | 2774 | 0.3 (0.2-0.5) | 5.8 (5.3-6.3) | 66.8 (64.3-69.3) |
| 2012 | 22 | 556 | 3045 | 0.3 (0.2-0.5) | 6.1 (5.6-6.6) | 71.1 (68.6-73.7) |
| 2013 | Suppressed | 44 | 254 | N/A | 0.5 (0.3-0.6) | 5.8 (5.1-6.6) |
| 2014 | 0 | 26 | 107 | N/A | 0.3 (0.2-0.4) | 2.4 (1.9-2.8) |
| 2015 | Suppressed | 22 | 123 | N/A | 0.2 (0.1-0.3) | 2.7 (2.2-3.1) |
| 2016 | Suppressed | 28 | 163 | N/A | 0.3 (0.2-0.4) | 3.4 (2.9-4) |
| 2017 | Suppressed | 44 | 214 | N/A | 0.4 (0.3-0.6) | 4.4 (3.8-5) |
| 2018 | Suppressed | 48 | 211 | N/A | 0.5 (0.4-0.7) | 4.2 (3.7-4.8) |
| 2019 | Suppressed | 53 | 246 | N/A | 0.6 (0.4-0.8) | 4.8 (4.2-5.4) |
| 2020 | Suppressed | 61 | 280 | N/A | 0.6 (0.5-0.8) | 5.4 (4.7-6) |
| **Overall** | 176 | 4076 | 21308 | 0.1 (0.1-0.1) | 2.2 (2.2-2.3) | 23.3 (23-23.6) |

Young Adult = 25-44 years; Middle Aged Adults = 45-64 years; Older Adults = 65 years and above; N/A = unreliable or suppressed
