# Supplementary material for: Emerging trends and disparities in cardiovascular, kidney, and diabetes-related mortality: A retrospective analysis of the wide-ranging online data for epidemiologic research database
Source: PLoS One. 2025 May 5;20(5):e0320670. doi: 10.1371/journal.pone.0320670 (PMC12052136; doi:10.1371/journal.pone.0320670)
Supplement: S6 Table — NH=non-Hispanic; N/A = unreliable or suppressed. (DOCX) [file pone.0320670.s006.docx]

**S6 Table. Cardiovascular-kidney metabolic syndrome -related Age-Adjusted Mortality Rates per 1,000,000, Stratified by Race in Adults in the United States from 1999 to 2020.**

| Deaths | | | | | | Age-Adjusted Rate (95% CI) | | | | |
| --- | --- | --- | --- | --- | --- | --- | --- | --- | --- | --- |
| Year | **NH White** | **NH Black or African American** | **NH American Indian or Alaska Native** | **Hispanic or Latino** | **NH Asian or Pacific Islander** | **NH White** | **NH Black or African American** | **NH American Indian or Alaska Native** | **Hispanic or Latino** | **NH Asian or Pacific Islander** |
| 1999 | 689 | 149 | Suppressed | 54 | 32 | 4.6 (4.3-5) | 10 (8.3-11.6) | N/A | 6 (4.5-7.9) | 7.8 (5.3-11.2) |
| 2000 | 792 | 153 | 11 | 72 | 30 | 5.3 (4.9-5.6) | 10 (8.4-11.6) | (6.7-25.7) | 7.5 (5.8-9.6) | 7.3 (4.9-10.6) |
| 2001 | 889 | 171 | 17 | 82 | 27 | 5.8 (5.5-6.2) | 11 (9.3-12.6) | (12.5-35.4) | 8.7 (6.9-10.9) | 6.5 (4.2-9.7) |
| 2002 | 914 | 191 | 16 | 71 | 31 | 5.9 (5.5-6.3) | 12.3 (10.5-14) | (10.5-32.1) | 6.8 (5.3-8.7) | 6.3 (4.2-9.1) |
| 2003 | 1067 | 229 | 15 | 96 | 36 | 6.9 (6.5-7.3) | 14.5 (12.6-16.4) | (9.9-31.9) | 9.3 (7.4-11.4) | 6.8 (4.7-9.6) |
| 2004 | 1153 | 184 | 14 | 122 | 43 | 7.3 (6.9-7.8) | 11.1 (9.5-12.8) | (9.5-30.4) | 10.7 (8.7-12.6) | 7.7 (5.5-10.5) |
| 2005 | 1199 | 240 | 18 | 160 | 44 | 7.5 (7.1-7.9) | 14.3 (12.4-16.1) | (12-34.2) | 13.6 (11.4-15.8) | 6.7 (4.8-9.1) |
| 2006 | 1137 | 237 | 16 | 140 | 46 | 7 (6.6-7.4) | 13.9 (12.1-15.7) | (7.5-22.9) | 10.8 (8.9-12.7) | 7.2 (5.2-9.8) |
| 2007 | 1115 | 245 | 18 | 120 | 42 | 6.8 (6.4-7.2) | 14.4 (12.6-16.3) | (12.4-35.2) | 9 (7.3-10.7) | 6.2 (4.4-8.4) |
| 2008 | 1113 | 228 | 16 | 126 | 47 | 6.7 (6.3-7.1) | 12.7 (11-14.4) | (8.3-25.5) | 9.2 (7.5-10.9) | 6.8 (5-9.2) |
| 2009 | 1124 | 210 | 25 | 148 | 46 | 6.6 (6.2-7) | 11.1 (9.6-12.7) | 20.2 (12.5-30.9) | 10.1 (8.4-11.7) | 6.5 (4.7-8.8) |
| 2010 | 1013 | 201 | 14 | 139 | 53 | 5.9 (5.5-6.3) | 10.6 (9.1-12.2) | (6.2-20.9) | 9.1 (7.5-10.7) | 6.7 (4.9-8.8) |
| 2011 | 2327 | 477 | 35 | 343 | 138 | 13.2 (12.7-13.8) | 24.8 (22.6-27.1) | 29 (19.8-40.9) | 21.1 (18.8-23.4) | 17.1 (14.2-20.1) |
| 2012 | 2492 | 541 | 35 | 398 | 150 | 13.9 (13.4-14.5) | 26.8 (24.5-29.1) | 32.7 (22.4-46.2) | 23.1 (20.8-25.5) | 17.4 (14.6-20.2) |
| 2013 | 192 | 43 | Suppressed | 49 | 11 | 1.1 (0.9-1.2) | 2 (1.5-2.8) | N/A | 2.9 (2.1-3.8) | (0.6-2.2) |
| 2014 | 88 | 26 | Suppressed | 11 | Suppressed | 0.5 (0.4-0.6) | 1.2 (0.8-1.8) | N/A | (0.3-1) | N/A |
| 2015 | 103 | 23 | Suppressed | 17 | Suppressed | 0.5 (0.4-0.6) | 1.1 (0.7-1.6) | N/A | (0.5-1.4) | N/A |
| 2016 | 144 | 26 | Suppressed | 16 | Suppressed | 0.7 (0.6-0.9) | 1.2 (0.8-1.8) | N/A | (0.5-1.3) | N/A |
| 2017 | 173 | 31 | Suppressed | 39 | 12 | 0.9 (0.7-1) | 1.2 (0.8-1.8) | N/A | 1.8 (1.3-2.5) | (0.5-1.8) |
| 2018 | 180 | 29 | Suppressed | 36 | 12 | 0.9 (0.8-1) | 1.2 (0.8-1.7) | N/A | 1.6 (1.1-2.2) | (0.5-1.8) |
| 2019 | 205 | 34 | Suppressed | 40 | 13 | 1 (0.9-1.1) | 1.4 (1-1.9) | N/A | 1.5 (1.1-2.1) | (0.5-1.6) |
| 2020 | 233 | 38 | Suppressed | 54 | 12 | 1.1 (1-1.3) | 1.4 (1-1.9) | N/A | 2.1 (1.6-2.7) | (0.5-1.5) |
| Overall | 18342 | 3706 | Suppressed | 2333 | 840 | 4.8 (4.7-4.9) | 8.6 (8.4-8.9) | N/A | 6.6 (6.4-6.9) | 4.7 (4.4-5) |

NH=non-Hispanic; N/A = unreliable or suppressed
