# Supplementary material for: Emerging trends and disparities in cardiovascular, kidney, and diabetes-related mortality: A retrospective analysis of the wide-ranging online data for epidemiologic research database
Source: PLoS One. 2025 May 5;20(5):e0320670. doi: 10.1371/journal.pone.0320670 (PMC12052136; doi:10.1371/journal.pone.0320670)
Supplement: S7 Table — (DOCX) [file pone.0320670.s007.docx]

**S7 Table. Cardiovascular-kidney metabolic syndrome -related Age-Adjusted Mortality Rates per 1,000,000, Stratified by State in Adults in the United States, 1999 to 2020.**

| State | Age-Adjusted Rate (95% CI) |
| --- | --- |
| Alabama | 4.8 (4.3-5.3) |
| Alaska | 6.9 (4.9-9.4) |
| Arizona | 2.7 (2.4-3) |
| Arkansas | 4 (3.4-4.5) |
| California | 7.7 (7.5-7.9) |
| Colorado | 4.3 (3.7-4.8) |
| Connecticut | 2.7 (2.3-3.1) |
| Delaware | 5 (3.9-6.3) |
| District of Columbia | 4.4 (3.1-6) |
| Florida | 2.3 (2.1-2.4) |
| Georgia | 3.1 (2.8-3.4) |
| Hawaii | 9.3 (8-10.5) |
| Idaho | 4.6 (3.7-5.5) |
| Illinois | 4.2 (3.9-4.5) |
| Indiana | 6.4 (5.9-7) |
| Iowa | 8.7 (8-9.5) |
| Kansas | 4.2 (3.6-4.8) |
| Kentucky | 5.8 (5.2-6.3) |
| Louisiana | 1.6 (1.3-1.9) |
| Maine | 6.2 (5.2-7.2) |
| Maryland | 4.5 (4.1-5) |
| Massachusetts | 2.5 (2.2-2.8) |
| Michigan | 4.1 (3.8-4.4) |
| Minnesota | 8.5 (7.9-9.1) |
| Mississippi | 4.2 (3.6-4.8) |
| Missouri | 5.9 (5.4-6.4) |
| Montana | 5.5 (4.4-6.7) |
| Nebraska | 8 (7-9.1) |
| Nevada | 0.9 (0.6-1.3) |
| New Hampshire | 4.8 (3.9-5.8) |
| New Jersey | 2.6 (2.3-2.9) |
| New Mexico | 3.1 (2.5-3.8) |
| New York | 2.8 (2.6-3) |
| North Carolina | 6.6 (6.2-7.1) |
| North Dakota | 11.6 (9.7-13.5) |
| Ohio | 9.9 (9.4-10.3) |
| Oklahoma | 7.2 (6.5-7.9) |
| Oregon | 6.6 (6-7.3) |
| Pennsylvania | 6.6 (6.3-7) |
| Rhode Island | 4.6 (3.7-5.7) |
| South Carolina | 5.8 (5.2-6.3) |
| South Dakota | 6.7 (5.4-8.2) |
| Tennessee | 7.8 (7.3-8.4) |
| Texas | 7.1 (6.8-7.4) |
| Utah | 2.8 (2.2-3.5) |
| Vermont | 6.4 (5-8.1) |
| Virginia | 4.5 (4.1-4.9) |
| Washington | 7.7 (7.1-8.2) |
| West Virginia | 9.5 (8.5-10.6) |
| Wisconsin | 5.2 (4.8-5.7) |
| Wyoming | 5.1 (3.6-6.9) |
| Overall | 5.4 (5.3-5.4) |
