# Supplementary material for: Emerging trends and disparities in cardiovascular, kidney, and diabetes-related mortality: A retrospective analysis of the wide-ranging online data for epidemiologic research database
Source: PLoS One. 2025 May 5;20(5):e0320670. doi: 10.1371/journal.pone.0320670 (PMC12052136; doi:10.1371/journal.pone.0320670)
Supplement: S8 Table — (DOCX) [file pone.0320670.s008.docx]

**S8 Table. Cardiovascular-kidney metabolic syndrome -related Age-Adjusted Mortality Rate per 1,000,000, Stratified by Census Region in Adults in the United States 1999-2020.**

| Census Region | Year | Age-Adjusted Rate (95% CI) |
| --- | --- | --- |
| Northeast | 1999 | 4.4 (3.7-5) |
| Northeast | 2000 | 5.5 (4.8-6.3) |
| Northeast | 2001 | 5.4 (4.7-6.1) |
| Northeast | 2002 | 6.1 (5.3-6.9) |
| Northeast | 2003 | 6.8 (6-7.7) |
| Northeast | 2004 | 6.6 (5.7-7.4) |
| Northeast | 2005 | 6.2 (5.4-7) |
| Northeast | 2006 | 5 (4.3-5.7) |
| Northeast | 2007 | 5.1 (4.4-5.8) |
| Northeast | 2008 | 4.1 (3.4-4.7) |
| Northeast | 2009 | 5.2 (4.5-5.9) |
| Northeast | 2010 | 4.4 (3.8-5.1) |
| Northeast | 2011 | 8.9 (8-9.8) |
| Northeast | 2012 | 10.8 (9.9-11.8) |
| Northeast | 2013 | 0.9 (0.6-1.2) |
| Northeast | 2014 | (0.2-0.5) |
| Northeast | 2015 | (0.3-0.7) |
| Northeast | 2016 | 0.6 (0.4-0.9) |
| Northeast | 2017 | 0.7 (0.5-1) |
| Northeast | 2018 | 0.5 (0.3-0.7) |
| Northeast | 2019 | 0.6 (0.4-0.9) |
| Northeast | 2020 | 1 (0.7-1.3) |
| Northeast | **Overall** | 3.9 (3.7-4) |
| Midwest | 1999 | 6.6 (5.8-7.4) |
| Midwest | 2000 | 7.9 (7-8.7) |
| Midwest | 2001 | 8.2 (7.3-9) |
| Midwest | 2002 | 8.6 (7.7-9.4) |
| Midwest | 2003 | 9.4 (8.4-10.3) |
| Midwest | 2004 | 9.9 (9-10.9) |
| Midwest | 2005 | 11 (10-11.9) |
| Midwest | 2006 | 10.1 (9.2-11) |
| Midwest | 2007 | 9.5 (8.6-10.4) |
| Midwest | 2008 | 8.8 (7.9-9.6) |
| Midwest | 2009 | 8.7 (7.9-9.5) |
| Midwest | 2010 | 7.5 (6.8-8.3) |
| Midwest | 2011 | 16.7 (15.5-17.8) |
| Midwest | 2012 | 18 (16.9-19.2) |
| Midwest | 2013 | 1.4 (1.1-1.7) |
| Midwest | 2014 | 0.6 (0.4-0.9) |
| Midwest | 2015 | 0.7 (0.5-0.9) |
| Midwest | 2016 | 0.8 (0.6-1.1) |
| Midwest | 2017 | 1.2 (0.9-1.5) |
| Midwest | 2018 | 0.9 (0.7-1.2) |
| Midwest | 2019 | 1.2 (0.9-1.5) |
| Midwest | 2020 | 1.2 (1-1.5) |
| Midwest | **Overall** | 6.4 (6.3-6.6) |
| South | 1999 | 5.1 (4.6-5.7) |
| South | 2000 | 5.5 (4.9-6.1) |
| South | 2001 | 6.7 (6.1-7.3) |
| South | 2002 | 5.9 (5.3-6.5) |
| South | 2003 | 7.4 (6.7-8) |
| South | 2004 | 7.4 (6.7-8) |
| South | 2005 | 8.4 (7.7-9.1) |
| South | 2006 | 7.9 (7.2-8.5) |
| South | 2007 | 7.1 (6.5-7.8) |
| South | 2008 | 7.4 (6.8-8) |
| South | 2009 | 6.9 (6.4-7.5) |
| South | 2010 | 6.2 (5.6-6.7) |
| South | 2011 | 13.8 (13-14.7) |
| South | 2012 | 13.7 (12.9-14.5) |
| South | 2013 | 1.1 (0.9-1.4) |
| South | 2014 | 0.6 (0.4-0.8) |
| South | 2015 | 0.5 (0.4-0.7) |
| South | 2016 | 0.7 (0.5-0.9) |
| South | 2017 | 0.8 (0.7-1) |
| South | 2018 | 1 (0.8-1.2) |
| South | 2019 | 1 (0.8-1.2) |
| South | 2020 | 1.1 (0.9-1.3) |
| South | **Overall** | 4.9 (4.8-5) |
| West | 1999 | 4.9 (4.2-5.6) |
| West | 2000 | 4.8 (4.1-5.5) |
| West | 2001 | 5.6 (4.8-6.3) |
| West | 2002 | 6.3 (5.5-7.1) |
| West | 2003 | 7.3 (6.5-8.2) |
| West | 2004 | 8.2 (7.3-9.1) |
| West | 2005 | 8.5 (7.6-9.4) |
| West | 2006 | 8.6 (7.7-9.5) |
| West | 2007 | 9 (8.1-10) |
| West | 2008 | 9.3 (8.4-10.3) |
| West | 2009 | 8.9 (8-9.8) |
| West | 2010 | 8.5 (7.6-9.3) |
| West | 2011 | 21.6 (20.3-22.9) |
| West | 2012 | 23 (21.7-24.4) |
| West | 2013 | 1.9 (1.5-2.3) |
| West | 2014 | 0.7 (0.5-1) |
| West | 2015 | 0.9 (0.6-1.2) |
| West | 2016 | 1.1 (0.8-1.4) |
| West | 2017 | 1.4 (1.1-1.8) |
| West | 2018 | 1.6 (1.3-2) |
| West | 2019 | 1.7 (1.4-2.1) |
| West | 2020 | 1.8 (1.5-2.2) |
| West | **Overall** | 6.3 (6.1-6.4) |
