# Supplementary material for: Emerging trends and disparities in cardiovascular, kidney, and diabetes-related mortality: A retrospective analysis of the wide-ranging online data for epidemiologic research database
Source: PLoS One. 2025 May 5;20(5):e0320670. doi: 10.1371/journal.pone.0320670 (PMC12052136; doi:10.1371/journal.pone.0320670)
Supplement: S9 Table — (DOCX) [file pone.0320670.s009.docx]

**S9 Table. Overall Cardiovascular-kidney metabolic syndrome –related Age-Adjusted Mortality Rates per 1,000,000, Stratified by Urbanization Status in Adults in the United States, 1999 to 2020.**

| Age-Adjusted Rate (95% CI) | | | |
| --- | --- | --- | --- |
| Year | **Large Metropolitan** | **Medium to small metropolitan** | **Rural/non-metropolitan** |
| 1999 | 4 (4.9-0.2) | 5.1 (6.4-0.3) | 5.9 (7.7-0.4) |
| 2000 | 4.2 (5-0.2) | 5.9 (7.3-0.3) | 7.4 (9.3-0.5) |
| 2001 | 4.8 (5.7-0.2) | 6.5 (7.9-0.4) | 7.9 (9.9-0.5) |
| 2002 | 4.7 (5.7-0.2) | 7.2 (8.7-0.4) | 7.5 (9.4-0.5) |
| 2003 | 5.5 (6.4-0.3) | 8.3 (9.8-0.4) | 9.3 (11.5-0.5) |
| 2004 | 6 (7-0.3) | 8.6 (10.2-0.4) | 8.7 (10.8-0.5) |
| 2005 | 6.2 (7.2-0.3) | 9.3 (10.9-0.4) | 10.1 (12.3-0.6) |
| 2006 | 5.9 (6.9-0.3) | 8.8 (10.4-0.4) | 8.7 (10.8-0.5) |
| 2007 | 5.6 (6.6-0.2) | 8.2 (9.6-0.4) | 9 (11-0.5) |
| 2008 | 5.5 (6.4-0.2) | 7.7 (9.1-0.4) | 9.1 (11.1-0.5) |
| 2009 | 5 (5.9-0.2) | 8 (9.4-0.4) | 9.7 (11.8-0.5) |
| 2010 | 5 (5.9-0.2) | 7 (8.4-0.3) | 7.4 (9.3-0.5) |
| 2011 | 13.1 (14.5-0.4) | 15.4 (17.3-0.5) | 15.7 (18.3-0.7) |
| 2012 | 13.9 (15.3-0.4) | 16.7 (18.6-0.5) | 16.7 (19.3-0.7) |
| 2013 | 1 (1.4-0.1) | 1.3 (1.9-0.1) | 0.8 (1.5-0.2) |
| 2014 | 0.4 (0.7-0.1) | 0.4 (0.8-0.1) | 0.3 (0.8-0.1) |
| 2015 | 0.4 (0.7-0.1) | 0.4 (0.8-0.1) | 0.6 (1.1-0.1) |
| 2016 | 0.6 (0.9-0.1) | 0.5 (0.9-0.1) | 0.6 (1.3-0.2) |
| 2017 | 0.8 (1.1-0.1) | 1 (1.5-0.1) | 0.6 (1.2-0.1) |
| 2018 | 0.8 (1.1-0.1) | 0.9 (1.4-0.1) | 0.8 (1.4-0.2) |
| 2019 | 0.8 (1.1-0.1) | 1.1 (1.6-0.1) | 1.1 (1.9-0.2) |
| 2020 | 0.8 (1.2-0.1) | 1.3 (1.9-0.1) | 1.1 (1.9-0.2) |
| Overall | 4.3 (4.5-0) | 6 (6.2-0.1) | 6.6 (7-0.1) |
