# Supplementary material for: Emerging trends and disparities in cardiovascular, kidney, and diabetes-related mortality: A retrospective analysis of the wide-ranging online data for epidemiologic research database
Source: PLoS One. 2025 May 5;20(5):e0320670. doi: 10.1371/journal.pone.0320670 (PMC12052136; doi:10.1371/journal.pone.0320670)
Supplement: S10 Table — (DOCX) [file pone.0320670.s010.docx]

**S10 Table. Overall Individual disease–related Age-Adjusted Mortality Rates per 100,000 in the United States, 1999 to 2020.**

| Age-Adjusted Rate (95% CI) | | | |
| --- | --- | --- | --- |
| Year | **Cardiovascular Disease** | **Chronic Renal Failure** | **Diabetes Mellitus Type-2** |
| 1999 | 541 (539.9-542.1) | 33.8 (33.5-34) | 21.5 (21.3-21.8) |
| 2000 | 526.4 (525.3-527.5) | 34.9 (34.6-35.2) | 23.5 (23.2-23.7) |
| 2001 | 509.6 (508.6-510.7) | 36 (35.8-36.3) | 25 (24.8-25.2) |
| 2002 | 500.2 (499.1-501.2) | 37 (36.7-37.3) | 26.7 (26.4-26.9) |
| 2003 | 482.8 (481.9-483.8) | 38.2 (37.9-38.5) | 27.6 (27.3-27.8) |
| 2004 | 453.9 (452.9-454.9) | 38.3 (38-38.6) | 28.1 (27.9-28.3) |
| 2005 | 441.8 (440.9-442.7) | 38.7 (38.4-38.9) | 29.8 (29.5-30) |
| 2006 | 415.5 (414.6-416.4) | 37.5 (37.2-37.8) | 29.7 (29.4-29.9) |
| 2007 | 397.9 (397.1-398.8) | 36.3 (36-36.6) | 30.1 (29.8-30.3) |
| 2008 | 388.6 (387.8-389.5) | 35 (34.8-35.3) | 30.6 (30.4-30.9) |
| 2009 | 369.5 (368.7-370.3) | 34 (33.8-34.3) | 30.4 (30.1-30.6) |
| 2010 | 363 (362.2-363.8) | 33.4 (33.1-33.6) | 30.6 (30.3-30.8) |
| 2011 | 352.3 (351.5-353.1) | 58.3 (58-58.7) | 30 (29.8-30.2) |
| 2012 | 345.7 (344.9-346.4) | 60.1 (59.8-60.4) | 29.9 (29.7-30.1) |
| 2013 | 343.6 (342.9-344.4) | 36.1 (35.8-36.3) | 30.4 (30.2-30.6) |
| 2014 | 338.9 (338.2-339.7) | 36.5 (36.2-36.7) | 29.9 (29.7-30.1) |
| 2015 | 343.3 (342.6-344.1) | 39 (38.7-39.2) | 31.9 (31.7-32.2) |
| 2016 | 338.3 (337.5-339) | 40.7 (40.5-41) | 36.1 (35.8-36.3) |
| 2017 | 338.2 (337.4-338.9) | 42 (41.8-42.3) | 39.2 (39-39.5) |
| 2018 | 334.7 (334-335.4) | 43 (42.7-43.2) | 40.6 (40.3-40.8) |
| 2019 | 330.8 (330.1-331.5) | 44 (43.7-44.2) | 42 (41.8-42.3) |
| 2020 | 346 (345.2-346.7) | 50.8 (50.5-51.1) | 57.7 (57.4-58) |
| Overall | 359.6 (358.8-360.3) | 53.9 (53.6-54.1) | 62.7 (62.4-63) |
